# Estrogen regulates miRNA expression: implication of estrogen receptor and miR-124/AKT2 in tumor growth and angiogenesis

**DOI:** 10.18632/oncotarget.9230

**Published:** 2016-05-09

**Authors:** Cheng-Fei Jiang, Dong-Mei Li, Zhu-Mei Shi, Lin Wang, Min-Min Liu, Xin Ge, Xue Liu, Ying-Chen Qian, Yi-Yang Wen, Lin-Lin Zhen, Jie Lin, Ling-Zhi Liu, Bing-Hua Jiang

**Affiliations:** ^1^ State Key Laboratory of Reproductive Medicine, Department of Pathology, Nanjing Medical University, Nanjing, China; ^2^ Department of Neurosurgery, The First Affiliated Hospital of Nanjing Medical University, Nanjing, China; ^3^ Huai'an First People's Hospital, Nanjing Medical University, Huai'an, China; ^4^ Faculty of Software, Fujian Normal University, Fuzhou, China; ^5^ Department of Pathology, Anatomy and Cell Biology, Thomas Jefferson University, Philadelphia, PA, USA

**Keywords:** estrogen, miR-124, AKT2, breast cancer, estrogen receptors

## Abstract

It is currently known that estrogen plays an important role in breast cancer (BC) development, but the underlying molecular mechanism remains to be elucidated. Accumulating evidence has revealed important roles of microRNAs in various kinds of human cancers, including BC. In this study, we found that among the microRNAs regulated by estrogen, miR-124 was the most prominent downregulated miRNA. miR-124 was downregulated by estradiol (E2) treatment in estrogen receptor (ER) positive BC cells, miR-124 overexpression suppressed cell proliferation, migration and invasion in BC cells; while the suppression of miR-124 using Anti-miR-124 inhibitor had opposite cellular functions. Under the E2 treatment, miR-124 had stronger effect to inhibit cellular functions in MCF7 cells than that in MDA-MB-231 cells. In addition, we identified that ERα, but not ERβ, was required for E2-induced miR-124 downregulation. Furthermore, AKT2, a known oncogene, was a novel direct target of miR-124. AKT2 expression levels were inversely correlated with miR-124 expression levels in human breast cancer specimens. AKT2 was overexpressed in BC specimens, and its expression levels were much higher in ERα positive cancer tissues than those ERα negative cancer tissues. Consistent with miR-124 suppression, E2 treatment increased AKT2 expression levels in MCF7 cells via ERα. Finally, overexpression of miR-124 in MCF7 cells significantly suppressed tumor growth and angiogenesis by targeting AKT2. Our results provide a mechanistic insight into a functional role of new ERα/miR-124/AKT2 signaling pathway in BC development. miR-124 and AKT2 may be used as biomarkers for ERα positive BC and therapeutic effect in the future.

## INTRODUCTION

Breast cancer (BC) is one of the most common gynecological malignancies and estrogen receptor α (ERα) is expressed in about 75% of diagnosed breast tumors (ERα positive) [1, 2]. Growing evidence manifests that exposure to estrogens (estrone and estradiol) is an important determinant of BC risk, and ERα blockers have been widely used in clinic to treat BC [3, 4]. However, the expression of estrogen receptor β (ERβ), the other subunit of estrogen receptor, is reduced during tumorigenesis and is important in terminal differentiation of breast epithelial cells [5, 6]. Estrogen influences numerous cell signal pathways by activing ERα which is essential for the proliferation of a large subset of breast tumors [7–9]. Some reports have shown that estrogen could interfere with repair of mutations by speeding up the cell division, which may cause the occurrence of BC [10, 11]. During the development of BC, estrogen functions as a tumor promotor by acting ER which binds to the specific DNA sequences in the promoter regions of downstream signaling molecules called estrogen response elements (EREs) [12, 13]. Even almost all the downstream genes of ER share similar sequences, however the transcriptional activation effects differ from each other, which causes multitudinous functions of ER in BC. Although the carcinogenic role of estrogen in BC has been extensively explored in the past, the mechanisms of estrogen in BC are not well understood.

microRNAs (miRNAs) are small (approx. 20-22 nt) noncoding RNAs which function as post-transcriptional regulators by negatively regulating the stability or translational efficiency of their target mRNAs [14, 15]. In recent years, numerous evidences have shown the vital roles of miRNAs in cancer occurrence and development, including in BC [16, 17]. For example, miR-21 which is overexpressed in multiple cancers and functions as an oncogene by targeting different tumor suppressor genes, is also upregulated in BC [18–20]. miR-125b is downregulated in invasive BC and is associated with poor survival rate by regulating its direct target Ets1 in BC patients [21, 22]. A few studies indicated miRNAs may participate in the carcinogenesis process of estrogen induced BC [23], however, little information is known about the role and mechanism of miRNAs regulated by estrogen and their downstream signaling pathways in BC.

In this study, we found that estrogen upregulated and downregulated the expression of certain miRNAs, and miR-124 was the most prominent downregulated miRNAs in response to estrogen treatment. In this study we aim to address the following questions: (1) whether estrogen suppresses miR-124 expression through ER, and which subunit of ER is required for estrogen-regulated miR-124 downregulation; (2)what is the role of miR-124 in ER positive and ER negative BC cell proliferation, migration and invasion with ER treatment; (3) what is/are functional target(s) of the miR-124; (4) whether the target(s) is/are regulated by estrogen in ER positive breast cancer cells; and (5) the role of miR-124 in regulating ER positive breast tumor growth and angiogenesis. The results of this study will shed light on how estrogen regulates BC development through regulating miR-124, and will be helpful for finding new biomarkers and/or therapeutic targets for ER positive BC.

## RESULTS

### Estradiol (E2) mediates levels of certain miRNAs, and miR-124 is the most prominently downregulated miRNA which is inhibited by E2 treatment in estrogen receptor (ER) positive BC cells

**Figure 1:**
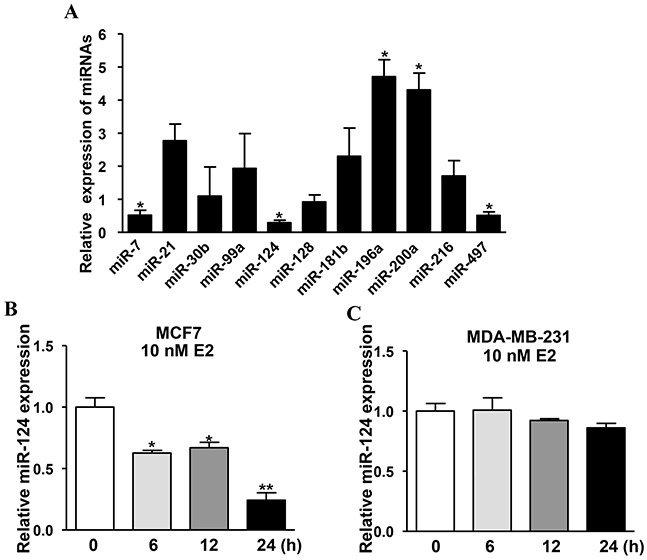
Estradiol (E2) mediates levels of certain miRNAs, and miR-124 is the most prominently downregulated miRNA which is inhibited by E2 treatment in estrogen receptor (ER) positive BC cells **A.** MCF7, the ER-positive BC cells were cultured estrogen-free medium for 72 h, then treated with 10 nM E2 or equal amount of ethyl alcohol (Eth) as solvent control. The expression levels of miRNAs were analyzed by qRT-PCR and U6 levels were used as internal control, and normalized to the values of Eth control. Data were presented as the means ± SD from three independent experiments with triple replicates per experiment. * indicates significant difference upon E2 treatment at *P* < 0.05. **B.** E2 treatment reduced miR-124 expression in MCF7 cells. Cells were cultured with E2 or Eth for 0, 6, 12 and 24 h. The relative miR-124 expression levels were analyzed as above. Data were presented as the means ± SD from three independent experiments with triple replicates per experiment. * and ** indicate significant difference under E2 treatment when compared to solvent control Eth with *P* < 0.05 and *P* < 0.01, respectively. **C.** E2 treatment had no effect on miR-124 expression in MDA-MB-231 cells. ER-negative BC cells MDA-MB-231 were treated and miR-124 was detected as above.

Although the carcinogenic effects of estrogen in breast tumor are generally accepted, the underlying molecular mechanisms remain elucidated. miRNAs are demonstratedto play significant roles in cancer occurrence and development by inhibiting the expression of their target genes at post-transcriptional levels. To explore whether estrogen regulates the expression of certain miRNAs in BC, the estrogen receptor positive breast cancer cells MCF7 were treated with 10nM E2 or isopyknic solvent ethyl alcohol (Eth) as control for 24h, then the expression levels of miRNAs were detected by qRT-PCR. Interestingly, several miRNAs were significantly upregulated (miR-196a and miR-200a), or downregulated (miR-7, miR-124 and miR-497) among which miR-124 was the most significantly downregulated miRNA (Figure 1A). Thus, we will test the role and mechanism of estrogen-suppressed miR-124 in BC development in this study. To test whether E2 inhibits miR-124 expression through the function of ER, the ER-positive and ER-negative BC cells MCF7 and MDA-MB-231 were treated with E2 or Eth at different time points and the results showed that miR-124 expression was downregulated at 6, 12 and 24 h post E2 treatment in MCF7 cells (Figure 1B), but not in MDA-MB-231 cells (Figure 1C), suggesting that ER is necessary for E2 to inhibit miR-124 expression.

### ERα, but not ERβ, is required for E2-suppressed miR-124 expression

**Figure 2:**
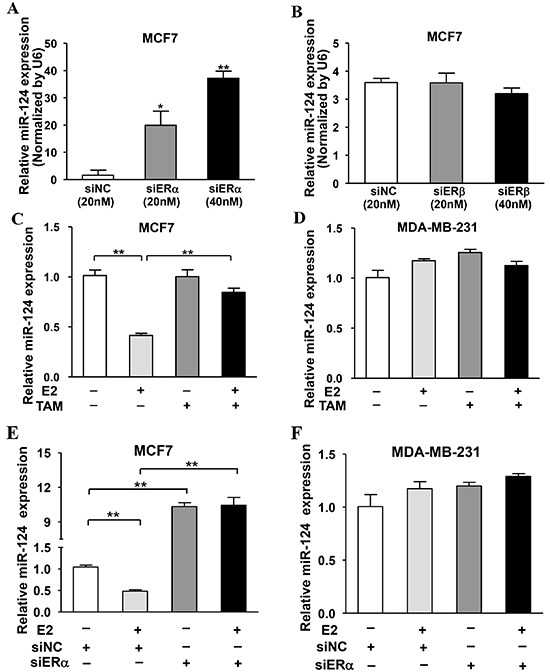
ERα, but not ERβ, was required for E2-suppressed miR-124 expression **A.** Knockdown of ERα in MCF7 cells induced miR-124 expression. **B.** ERβ silencing had no effect on miR-124 expression. MCF7 cells were transfected with different dose of ERα siRNAs, ERβ siRNAs or negative control siRNAs (siNC). After 72 h, the relative expression levels of miR-124 were analyzed by qRT-PCR and normalized to U6 expression levels. Data were presented as the means ± SD from three independent experiments with triple replicates per experiment. * and ** indicate significant difference compared to control with *P* < 0.05 and *P* < 0.01, respectively. **C.** E2 treatment decreased miR-124 expression, which was restored by tamoxifen (TAM) treatment. MCF7 cells were cultured in estrogen-free medium and treated without or with 10 nM E2 and 100 nM TAM for 24 h. The expression of miR-124 was detected as above. Data were presented as means ± SD from three independent experiments with triple replicates per experiment. ** indicates significant difference between two groups at *P* < 0.01. **D.** E2 and TAM had no effect on miR-124 expression. MDA-MB-231 cells were treated and miR-124 was analyzed as above. **E.** Knockdown of ERα recovered E2-suppressed miR-124 levels in MCF7 cells. MCF7 cells were cultured as above, then transfected with siERα or siNC for 24 h. Cells were treated with or without 10 nM E2 for 24 h and the expression of miR-124 were detected as above. Data were presented as the means ± SD from three independent experiments with triple replicates per experiment. ** indicates significant difference between two groups at *P* < 0.01. **F.** E2 treatment and knockdown of ERα showed no effect on miR-124 expression in MDA-MB-231 cells.

It is well known that ER is composed by two subunits ERα and ERβ. To further determine which subunit of ER is responsible for the downregulation of miR-124 expression, MCF7 cells were transfected with siRNAs against ERα, ERβ or negative control (siNC) to knock down the expression of ERα and ERβ in the cells, respectively. The results showed that the silence of ERα significantly inhibited miR-124 expression in a dose-dependent manner (Figure 2A). However, there was no effect of ERβ knockdown on miR-124 expression (Figure 2B), indicating that ERα, but not ERβ, is involved in regulating miR-124 expression. To further confirm the role of E2 and ERα in mediating miR-124 expression upon E2 treatment, we found that E2 decreased miR-124 levels in MCF7 cells, whereas the estrogen antagonist tamoxifen (TAM) restored miR-124 expression (Figure 2C). E2 or TAM treatment had no effect on miR-124 expression in MDA-MB-231 cells (Figure 2D). Similarly, knockdown of ERα recovered E2-suppressed miR-124 levels in MCF7 cells, but not in MDA-MB-231 cells (Figure 2E and 2F), demonstrating that miR-124 is regulated by E2 via ERα.

### Downregulation of endogenous miR-124 levels promotes cell proliferation, migration and invasion

**Figure 3:**
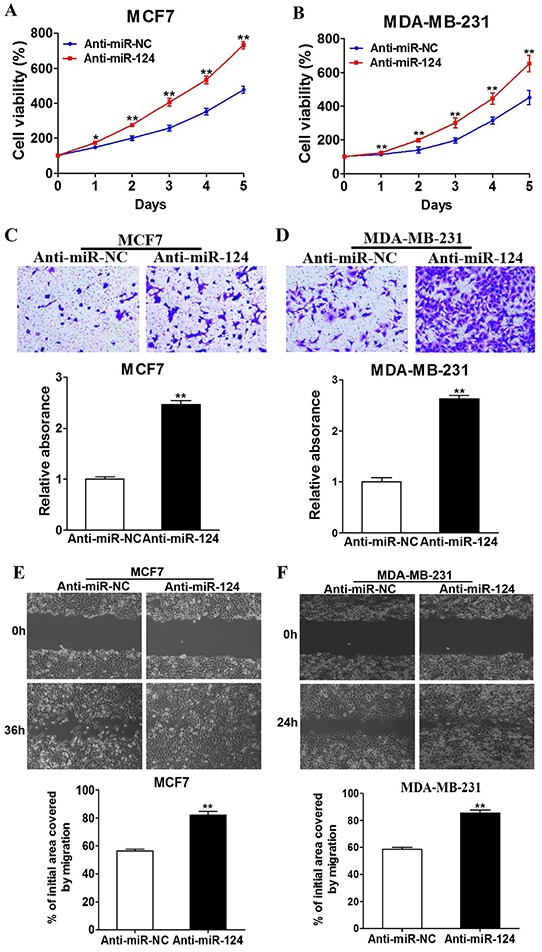
Downregulation of endogenous miR-124 promotes the proliferation, migration and invasion of BC cells **A. and B.** MCF7 and MDA-MB-231 cells were transfected with anti-miR-124 inhibitor (Anti-miR-124), or control anti-sense RNA (anti-miR-NC). Cell Counting Kit-8 (CCK-8) was used to detect cell vitality every 24 h, and the results were presented as the means± SD from three independent experiments performed in quintuple. * and ** indicate significant difference compared to anti-miR-NC with *P* < 0.05 and *P* < 0.01, respectively. **C. and D.** The treated cells were used to perform wound healing assay to estimate the ability of cell migration. A sterile 10 μl pipette tip was used to scratch the cells to form a wound when the different treated cells were cultured to 90% confluence. The wound gaps were photographed (top) and measured (bottom). Data were presented as the means±SD. ** indicates significant difference between two groups at *P* < 0.01 E. and F. The cells above were used to perform Matrigel invasion assay as described. After incubation for 24 h, the cells in the bottom of the invasion chamber were photographed and the acetic acid-eluted solution was quantified using a standard microplate reader at 570 nm. Data were presented as the means ± SD from three independent experiments with triple replicates per experiment. ** indicates significant difference between two groups at P < 0.01.

To identify whether endogenous miR-124 affects tumor progression, MCF7 and MDA-MB-231 cells were transfected with miR-124 inhibitor (Anti-miR-124) or control anti-sense RNA (Anti-miR-NC). Successful inhibition of endogenous miR-124 expression was confirmed by qRT-PCR (Supplementary Figure S1C–S1D). The inhibition of miR-124 remarkably increased cell growth, migration, and invasion of the breast cancer cells (Figure 3A-3F), demonstrating the potential important role of endogenous miR-124 in BC development. This result also indicates the downregulation of miR-124 induced by estrogen is involved in estrogen-mediated BC progression.

### miR-124 overexpression inhibits E2-induced cell proliferation, migration and invasion in ER-positive BC cells

**Figure 4:**
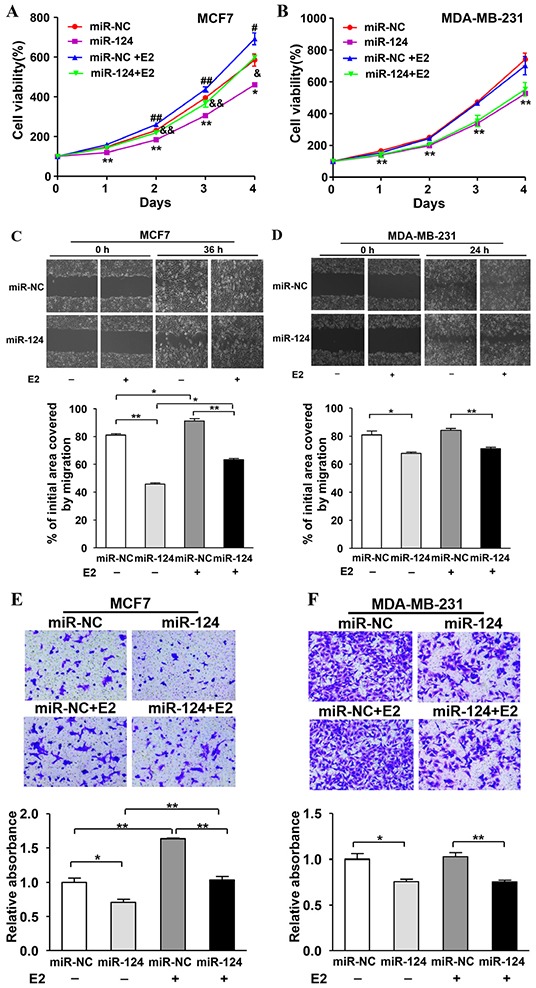
miR-124 overexpression inhibits E2-induced cell proliferation, migration and invasion in ER-positive BC cells The estrogen-positive and -negative cell lines MCF7 and MDA-MB-231 were infected with lentivirus carrying miR-124 or miR-NC to establish stable cell lines. **A.** MCF7/miR-124 and MCF7/miR-NC stable cells were plated at 2,000 cells/well in 96-well plates. After adherence, cells were treated with E2 or Eth solvent. Cell Counting Kit-8 (CCK-8) was used to detect cell vitality every 24 h and the results were presented as the means± SD from three independent experiments performed in quintuple. * and ** indicate significant difference compared to miR-NC without E2 treatment group with *P* < 0.05 and *P* < 0.01, respectively; # and ## indicate significant difference upon E2 treatment with *P* < 0.05 and *P* < 0.01, respectively; & and && indicate significant difference when compared to miR-NC with E2 treatment group with *P* < 0.05 and *P* < 0.01, respectively. **B.** MDA-MB-231/miR-124 and MDA-MB-231/miR-NC stable cells were treated and analyzed as above. ** indicates significant difference compared to miR-NC without E2 treatment group at *P* < 0.01. **C. and D.** The stable cells above with or without E2 treatment were used to perform wound healing assay to estimate the ability of cell migration. A sterile 10 μl pipette tip was used to scratch the cells to form a wound when the different treated cells were cultured to 90% confluence. The wound gaps were photographed (top) and measured (bottom). Data were presented as the means± SD. * and ** indicate significant difference between two groups with *P* < 0.05 and *P* < 0.01, respectively. **E. and F.** The stable cells above were used to perform Matrigel invasion assay as described. In addition, the E2 or Eth solvent was mixed into the medium respectively in the lower chamber. After incubation for 24 h, the cells in the bottom of the invasion chamber were photographed and the acetic acid-eluted solution was quantified using a standard microplate reader at 570 nm. Data were presented as the means ± SD from three independent experiments with triple replicates per experiment. * and ** indicate significant difference between two groups with P < 0.05 and P < 0.01, respectively.

To further study the role of miR-124 in E2-promoted BC development, MCF7 and MDA-MB-231 cells were transduced by lentivirus carrying miR-124 or negative control (miR-NC), and the stable cell lines were obtained by puromycin selection. We found that E2 treatment significantly increased cell proliferation, whereas forced expression of miR-124 attenuated E2-induced cell proliferation in MCF7 cells. In addition, miR-124 overexpression also decreased cell growth without E2 treatment (Figure 4A). Similarly, forced expression of miR-124 markedly suppressed E2 treatment-promoted cell migration and invasion in MCF7, the ER-positive BC cells (Figure 4C and 4E). On the contrary, E2 treatment had no effect on cell proliferation, migration and invasion in MDA-MB-231, the ER-negative BC cells. However, overexpression of miR-124 attenuated cell proliferation, migration and invasion when compared to miR-NC group in MDA-MB-231 cells (Figure 4B, 4D and 4F). These results show that miR-124 acts as a tumor suppressor toinhibittumor development by attenuating cell viability, migration and invasion in both ERα-positive and ERα-negative BC cells. More importantly, miR-124 plays an important role in ERα-positive BC cells to suppress the estrogen-induced cell viability, migration and invasion, demonstrating a novel role of miR-124 in E2-regulated BC, which accounts for about 75% of diagnosed breast tumors.

### miR-124 directly targets and inhibits AKT2 expression, miR-124 levels inversely correlates with AKT2 expression levels in ERα-positive BC tissues

**Figure 5:**
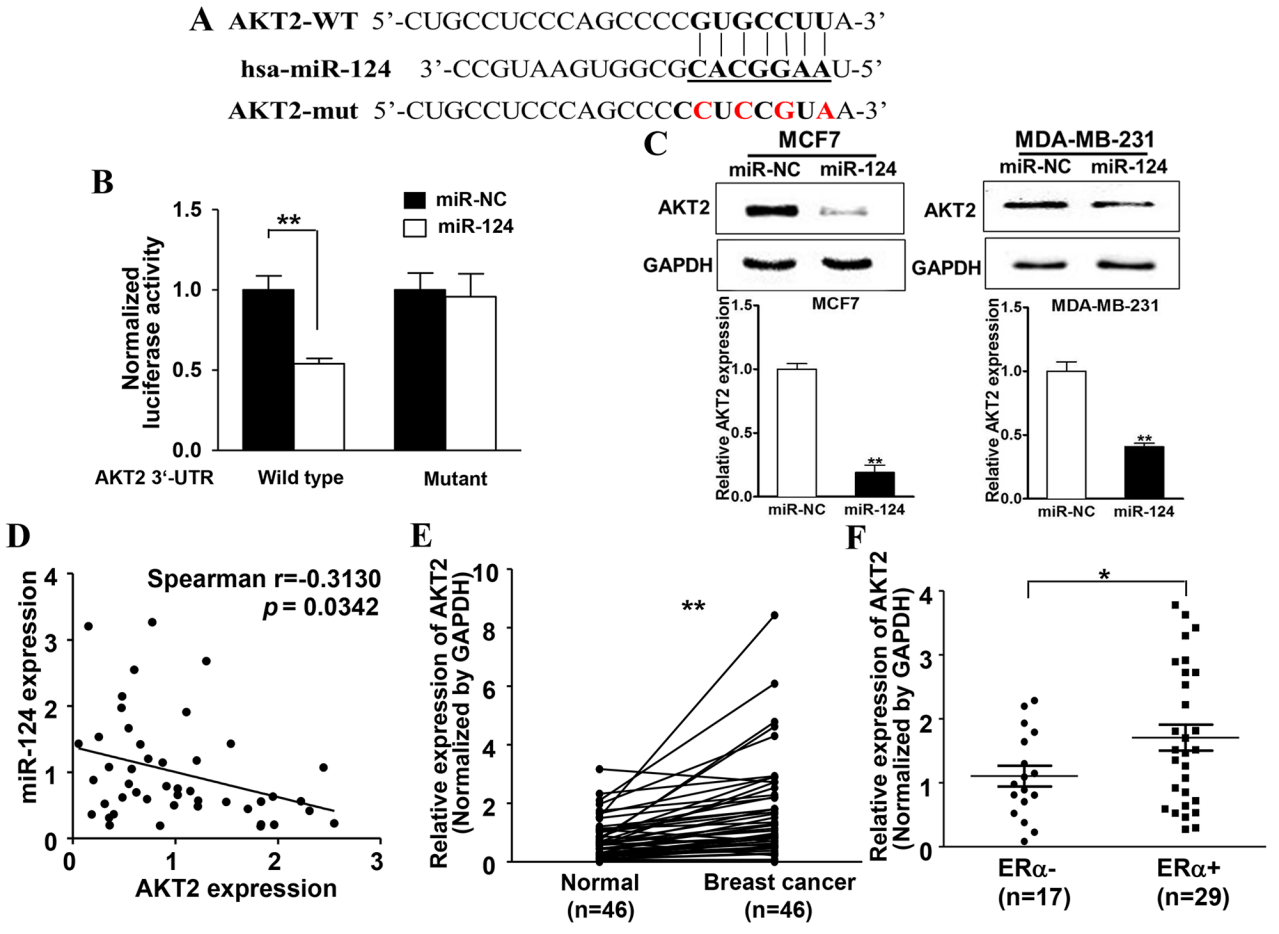
miR-124 directly targets and inhibits AKT2 expression, miR-124 levels inversely correlates with AKT2 expression levels in ERα-positive BC tissues **A.** Putative seed-matching sites (in bold) or mutant sites (red) between miR-124 and 3′-UTR of AKT2. **B.** Luciferase reporter assay was performed using MDA-MB-231 cells to detect the relative luciferase activities of WT and mut AKT2 reporters. Renilla luciferase vector was used as an internal control. Data were presented as the means± SD from three independent experiments with triple replicates per experiment. ** indicates significant difference compared to control at *P* < 0.01. **C.** The expression of AKT2 and GAPDH was determined using immunoblotting in MCF7 and MDA-MB-231 cells overexpressing miR-124 and miR-NC. The densities of AKT2 were quantified by ImageJ software and GAPDH levels were used as internal control, and normalized to the values of Eth control. Data were presented as the means ± SD from three independent experiments with triple replicates per experiment. ** indicates significant difference compared to control at *P* < 0.01. **D.** Spearman's correlation analysis was used to determine the correlation between the expression levels of AKT2 and miR-124 in human BC specimens (n=46). Data were presented as the means ± SD from three independent experiments with triple replicates per experiment. **E.** The expression levels of AKT2 in adjacent normal tissues and human BC specimens were determined by qRT-PCR, and the fold changes were obtained from the ratio of AKT2 to GAPDH levels. Data were presented as mean from three independent experiments with triple replicates per experiment. ** indicates significant difference comparing normal tissues at *P* < 0.01. **F.** The relative AKT2 expression levels of BC tumors were analyzed according to ERα status (ERα-negative, n=17; ERα–positive, n =29). Data were presented as mean from three independent experiments with triple replicates per experiment. * indicates significant difference comparing ERα-negative and ERα–positive tumors at *P* < 0.05.

To fully understand the mechanisms of miR-124 in suppressing E2 induced BC development, TargetScan search program was used to predict the targets of miR-124. We found AKT2, a well-known oncogene, could be a potential target of miR-124. The putative binding sites between miR-124 and 3′-UTR of miR-124 were shown in Figure 4A. To explore whether miR-124 directly targets the 3′-UTR of AKT2, we constructed luciferase reporter plasmids containing the putative wild-type binding sites (WT) and seed sequence mutant sites (mut) at 3′-UTR of AKT2 (Figure 5A) and verified by sequencing. Overexpression of miR-124 in MDA-MB-231 cells decreased luciferase activity of wild type reporter to 50%, suggesting that miR-124 may inhibit the 3′-UTR function of AKT2. To test whether miR-124 specifically inhibits AKT2 by binding its seed sequence, we also mutated the miR-124 binding site in the reporter construct (Mutant). Forced expression of miR-124 did not affect the transcriptional activation of mutant AKT2 3′-UTR (Figure 5B). In addition, stable cells of MCF7 and MDA-MB-231 overexpressing miR-124 showed decreased AKT2 expression at protein level (Figure 5C). These results indicate that AKT2 is a direct target of miR-124 by binding to the seed sequence. Furthermore, Spearman's rank correlation analysis showed an inverse correlation between expression levels of AKT2 and miR-124 in human BC specimens (Spearman's correlation r=−0.3130, Figure 5D). These results show the negative correlation between the expression levels of miR-124 and its target AKT in human BC tissues.

Next, to determine the expression levels of AKT2 in clinical BC specimens and adjacent normal tissues, qRT-PCR assay was performed to detect AKT2 expression levels in tissues, then analyzed by densitometric measurement and normalized to the GAPDH expression levels. Compared to the adjacent normal tissues, BC tissues (n = 46) showed significantly higher levels of AKT2 (*P* < 0.01, Figure 5E). And for the first time, we found that ERα-positive BC tissues showed higher AKT2 expression levels than ERα-negative BC tissues (*P* < 0.05, Figure 5F), suggesting that AKT2 may be a potential biomarker for BC detection and has significance to differentiate ERα-positive BC in clinical tissue samples.

### Forced expression of AKT2 reverses miR-124-suppressed cell proliferation, migration and invasion

**Figure 6:**
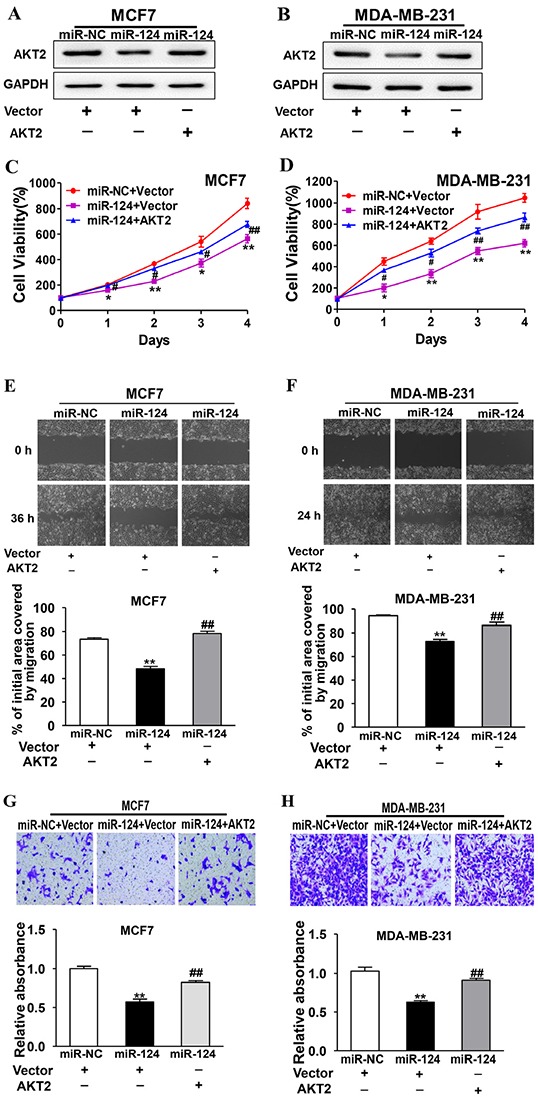
Forced expression of AKT2 reverses miR-124-suppressed cell proliferation, migration and invasion miR-124- or miR-NC-overexpressing cells were transfected with vector or AKT2 cDNA without 3′-UTR. **A. and B.** The expression levels of AKT2 and GAPDH were determined by immunoblotting after 48 h. **C. and D.** Cell viability was detected using CCK-8 assay. Data were presented as the means ± SD from three independent experiments with triple replicates per experiment. * and ** indicate significant difference compared to miR-NC+vector group with *P* < 0.05 and *P* < 0.01, respectively. # and ## indicate significant difference compared to miR-124+vector group with *P* < 0.05 and *P* < 0.01, respectively. **E. and F.** Cells were treated and wound healing assay was performed as above. Data were presented as the means ± SD from three independent experiments with triple replicates per experiment. ** indicates significant difference compared to miR-NC+vector group at *P* < 0.01; ## indicates significant difference compared to miR-124+vector group at *P* < 0.01. **G. and H.** Transwell invasion assay was performed as above using control cells and cells overexpressing miR-124 with or without AKT2 overexpression. Data were presented as the means ± SD from three independent experiments with triple replicates per experiment. ** indicates significant difference compared to miR-NC+vector group at P < 0.01; ## indicates significant difference compared to miR-124+vector group at P < 0.01.

To determine whether miR-124 inhibits BC development through its target AKT2, the stable cells of MCF7 and MDA-MB-231 cells overexpressing miR-124 were transfected with AKT2 cDNA without 3′-UTR. The immunoblotting results confirmed the effect of AKT2 cDNA transfection (Figure 6A and 6B). Overexpression of AKT2 was sufficient to reverse miR-124-inhibited cell proliferation, migration and invasion in both MCF7 (Figure 6C, 6E and 6G) and MDA-MB-231 cells (Figure 6D, 6F and 6H), suggesting that the inhibitory effect of miR-124 in human BC cells is via the function of its target AKT2.

### ERα is required for E2 upregulated-AKT2 expression, which can be inhibited by miR-124 in ERα-positive BC cells

**Figure 7:**
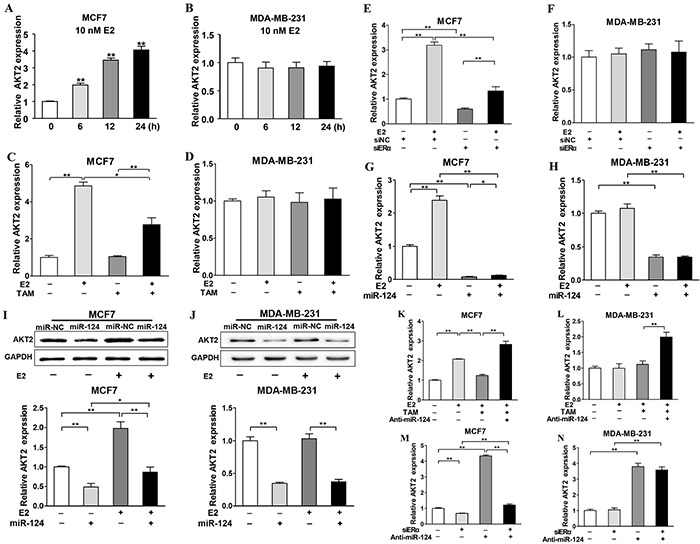
ERα is required for E2 upregulated-AKT2 expression, which can be inhibited by miR-124 in ERα-positive BC cells **A. and B.** MCF7 and MDA-MB-231 cells were cultured as above and treated with E2 for 0, 6, 12 and 24 h. The relative AKT2 expression of each group was analyzed by qRT-PCR and represented the ratio to control group. Data were presented as the means ± SD from three independent experiments with triple replicates per experiment. ** indicates significant difference compared to control at *P* < 0.01. **C. and D.** TAM was used as the E2 antagonist and the expression levels of AKT2 were analyzed by qRT-PCR. Data were presented as the means ± SD from three independent experiments with triple replicates per experiment. * and ** indicate significant difference between two groups with *P* < 0.05 and *P* < 0.01, respectively. **E. and F.** Cells were cultured and treated as in Figure 2E, the expression levels of AKT2 were analyzed as above. Data were presented as the means ± SD from three independent experiments with triple replicates per experiment. ** indicates significant difference between two groups at *P* < 0.01. **G. and H.** The cells were cultured as above and the expression levels of AKT2 were determined by qRT-PCR in miR-124- and miR-NC-overexpressing cells without or with E2 treatment for 24 h using GAPDH levels as internal control, and normalized to the value of Eth control. Data were presented as the means ± SD from three independent experiments with triple replicates per experiment. * and ** indicate significant differences between two groups with *P* < 0.05 and *P* < 0.01, respectively. **I. and J.** The expression levels of AKT2 and GAPDH were determined by immunoblotting in miR-124- and miR-NC-overexpressing cells without or with E2 treatment for 48 h. The densities of AKT2 were quantified by Image J software and GAPDH levels were used as internal control, and normalized to the values of Eth control. Data were presented as the means ± SD from three independent experiments with triple replicates per experiment. * and ** indicate significant differences between two groups with *P* < 0.05 and *P* < 0.01, respectively. **K. and L.** MCF7 and MDA-MB-231 cells were cultured as above and transfected with siRNAs, and divided in four groups including siNC+Anti-miR-NC, siERα+Anti-miR-NC, siNC+Anti-miR-124, siERα+ Anti-miR-124 group. After 24 h, the expression levels of AKT2 were determined by qRT-PCR using GAPDH levels as internal control, and normalized to the values of siNC+Anti-miR-NC group. Data were presented as the means ± SD from three independent experiments with triple replicates per experiment. ** indicates significant difference between two groups at *P* < 0.01 **M. and N.** Cells were cultured as above and transfected with Anti-miR-NC or Anti-miR-124. After 24 h, the cells were treated with or without 10 nM E2 and 100nM TAM for 24 h. The expression levels of AKT2 were determined by qRT-PCR using GAPDH levels as internal control, and normalized to the values of Eth+Anti-miR-NC group. Data were presented as the means ± SD from three independent experiments with triple replicates per experiment. ** indicates significant difference between two groups at *P* < 0.01.

Our previous results demonstrate that E2 regulates miR-124 expression in ERα-positive cells, but not in ERα-negative cells. To further study whether E2 affects expression levels of AKT2, the target of miR-124, we found that E2 treatment promoted AKT2 expression in MCF7 cells (Figure 7A), but not in MDA-MB-231 cells (Figure 7B). E2 treatment induced AKT2 expression to 4.5-fold, whereas TAM treatment decreased E2-induced AKT2 levels by 50% which was still higher than that in cells without E2 treatment (Figure 7C). On the contrary, E2 and TAM showed no effect on AKT2 expression in MDA-MB-231 cells, which is consistent with our previous results showing that E2 and TAM did not regulate miR-124 levels in MDA-MB-231 cells (Figure 7D). Furthermore, without E2 treatment, knockdown of ERα significantly increased AKT2 expression levels (*P* < 0.05) in MCF7 cells. Although the expression levels of AKT2 are higher in MCF7 cells under the treatment of E2 combination with ERα knockdown when compared to the cells ERα knockdown without E2 treatment, ERα is necessary for E2-induced AKT2 expression. However, neither E2 treatment nor ERα knockdown showed effect on AKT2 expression in MDA-MB-231 cells (Figure 7E and 7F). Although E2 treatment significantly induced AKT2 expression in MCF7 cells, but not in MDA-MB-231 cells, forced expression of miR-124 suppressed AKT2 expression with or without E2 treatment in both cells (Figure 7G–7J). In addition, silence of miR-124 by siRNAs reversed the AKT2 suppression caused by the interference of ERα in ER positive BC cells, and our result indicated high correlation between ERα and AKT2 levels via miR-124 levels (Figure 7K–7M).

### Overexpression of miR-124 inhibits tumor growth and angiogenesis

**Figure 8:**
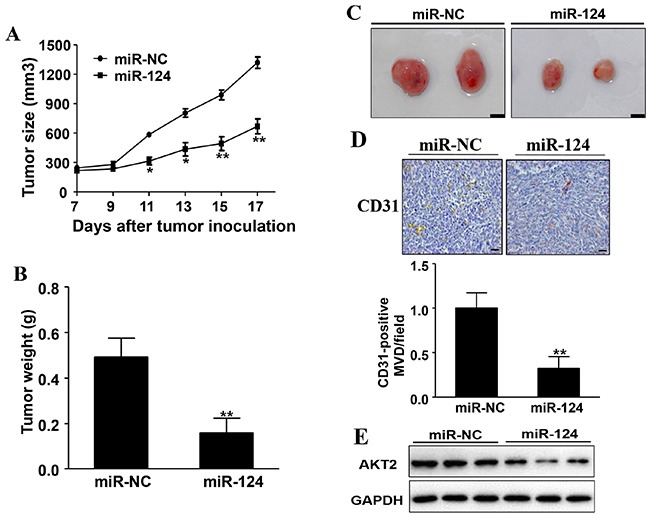
Overexpression of miR-124 inhibits tumor growth and angiogenesis **A.** MCF7/miR-124 and MCF7/miR-NC cells were dispersed in 100 μl of serum-free DMEM medium and were subcutaneously injected into both sides of posterior flank of the nude mice (n=4). Tumors were measured every two days since they were apparently seen and the volumes were calculated using the following formula: volume = 0.5 × Length × Width^2^. * and ** indicate significant difference compared to miR-NC group with *P* < 0.05 and *P* < 0.01, respectively. **B.** The tumor was excised and weighed after 17 days. Data were presented as the means ± SD. ** indicates significant difference compared to miR-NC group at *P* < 0.01. **C.** The representative pictures of trimmed tumors (Bar= 10mm). **D.** The expression levels of CD31 were analyzed in tumor tissues by immunohistochemistry. The density of CD31 levels was quantified by ImageJ software. Magnification, ×200, Scale bar, 20 μm. **E.** The whole protein was extracted from xenografts and subjected to immunoblotting assay for AKT2 expression. GAPDH expression was served as an internal control.

Finally, to further investigate the role of miR-124 in breast tumor growth and angiogenesis *in vivo*, ectopic transplantation model of human BC in nude mice was employed. MCF7 cells overexpressing miR-124 or miR-NC were subcutaneously injected into both posterior flanks of nude mice. Tumor volumes were monitored every two days when they were palpable during the tumor inoculation period. Compared to miR-124 group, the tumor size of miR-NC group was significantly increased by Day 11 (*P* < 0.05) and grew more and more quickly (Figure 8A). Nude mice were sacrificed 17 days after implantation and xenografts were trimmed out. The tumor size of miR-124 group was smaller than that of control group (Figure 8C). Consistent with tumor size, the tumor weight of miR-124 group was 31% of control group (Figure 8B).

Moreover, tumors from MCF7/miR-124 group showed decreased number of microvessels with CD31 positive staining by 68% when compared to control group (Figure 8D). In agreement with the results *in vitro*, overexpression of miR-124 suppressed AKT2 expression, in tumor tissues (Figure 8E). These results suggest that miR-124 suppresses tumorigenesis and angiogenesis of human ERα-positive BC in nude mice, and AKT2 is an important target involved in this process.

## DISCUSSION

Estrogen has been reported to play an important role in tumor occurrence and development during the past several decades [24]. Estrogen always acts as a carcinogenic factor by multiple aspects such as promoting cell proliferation, metastasis and drug-resistance of BC cells [3]. The effects of estrogen in BC have been widely studied; however, the underlying molecular mechanisms of estrogen in BC are still unclear. miRNAs are endogenous small non-coding RNAs, and play critical roles in cell growth, differentiation, apoptosis and tumourigenesis by negative regulating the mRNAs of oncogenes or tumor-suppressor genes [25, 26]. Some studies indicatethat miRNAs may play important roles in estrogen induced BC [27, 28]. It was showed that miR-515-5p was downregulated by estrogen treatment in ER positive BC cells to regulate the BC cell proliferation through its target SK1 [1]. ERα upregulated c-Myc expression which can downregulate ERα as a negative autoregulatory feedback loop [29]. However, there is still lack of the knowledge about the roles of estrogen-regulated miRNAs in BC development. In this study, we demonstrated that E2 could upregulate or downregulate certain miRNAs, and miR-124 was the most prominently downregulated miRNA which is regulated by E2 treatment in ER-positive BC cells. miR-124 has been reported to act as a tumor suppresser in many cancers including BC [30–32]. In this study, we discovered that estrogen-regulated miR-124 plays an important role in BC cell proliferation, migration, invasion, tumor growth and angiogenesis, and also identified that miR-124 suppression caused by E2 in ER-positive BC cells is through ERα instead of ERβ. Some reports indicated that ERα instead of ERβ interacts with and suppresses Drosha activity through which could downregulate numbers of miRNAs in ER-positive BC cells that were similar to our data [33], however, the in-depth mechanisms of downregulation of miR-124 induced by estrogen remains to be elucidated.

Our previous studies have showed that miR-124 could govern glioma growth and angiogenesis and enhance chemosensitivity by targeting R-Ras and N-Ras [34]. It was also reported that miR-124 inhibited cellular proliferation and invasion by targeting Ets-1 in breast cancer cells [35]. Although there were several identified targets of miR-124 which participated in miR-124 mediated BC development and progression, the researches about the functions and mechanisms of estrogen downregulated miR-124 in ER positive BC cells are still scanty. It has been reported that the activated AKT2 phosphorylates ERα and promotes the transcriptional activity of ERα [36, 37]. Interestingly, ERα can also activate AKT2 although the underlined mechanism is still unknown [36]. There is a correlation between ERα and AKT2. AKT2 (v-AKT murine thymoma viral oncogene homologue 2), an isoform of AKT family, is a significant member of the the PI3K/AKT pathway [38, 39]. Increasing studies demonstrate the important role of AKT2 in cancers as an oncogene [40] which is closely associated to tumor aggressiveness by enhancing the survival, migration and invasion of cancer cells primarily [36, 41], and overexpress in many human tumors including BC and ovarian cancer [42]. In this study, we discovered that AKT2 was a new direct target of miR-124, which was confirmed by clinical BC specimens showing that miR-124 levels inversely correlate with AKT2 expression levels. AKT2 was shown to be upregulated in ERα-positive BC tissues. miR-124 may act as a tumor suppressor to inhibit BC development by targeting AKT2, and ERα is required for E2 upregulated-AKT2 expression, which can bereversedby miR-124 in ERα-positive BC cells. Our results demonstrate a key role of a new regulatory circuitof E2/ERα/miR-124/AKT2 in mediating tumor angiogenesis and cancer progression in BC.

In summary, we have demonstrated that miR-124 is one of the miRNAs which are regulated by estrogen levels. The downregulation of miR-124 by E2 in ER-positive BC cells is through ERα but not ERβ, which will broaden our understanding the mechanisms of estrogen-modulated BC. Besides, we identified AKT2 as a novel target of miR-124, which is upregulated in clinical BC specimens, which may be used to differentiate ERα-positive and –negative BC. Similar to E2-downregulated miR-124, E2 induces AKT2 expression through ERα. The new regulatory circuit of E2/ERα/miR-124/AKT2 in BC plays an important role in BC tumorigenesis and development, which will provide potential novel biomarkers and targets for the diagnosis and treatment of BC.

## MATERIALS AND METHODS

### Human tissue samples

Human BC samples and adjacent normal tissues were obtained from patients in the First affiliated hospital of Anhui Medical University, Hefei, China and the Huai'an First People's Hospital, Nanjing Medical University. Tissue samples were collected at surgery, immediately frozen in liquid nitrogen and stored until total RNAs or proteins were extracted. All experiments were approved by the ethics committee of Anhui Medical University and Nanjing Medical University.

### Cell culture

BC cell lines MCF7 and MDA-MB-231 were maintained in Dulbecco's modified Eagle's medium (DMEM) supplemented with 10% fetal bovine serum (FBS), 100 units of penicillin/mL and 100 ng of streptomycin/mL. Human embryonic kidney 293T(HEK-293T) cells were cultured in DMEM supplemented with 10% FBS, penicillin(100U/ml), streptomycin(100ng/ml) and 2 mmol/mL glutamine. All cell lines were maintained in a 37°C incubator with 5% CO_2_. Before treatment with estradiol (17β-estradiol, E2; Sigma-Aldrich Ltd.), ethanol solvent (Eth, used as negative control), or tamoxifen (Sigma), cells were maintained for 3 days in DMEM without phenol red (Gibco) supplemented with 10% double charcoal–stripped FCS (Gibco) and pen/strep at 37°C with 5% CO_2_. On the day of treatment, media was changed to DMEM without phenol red (Gibco) supplemented with 10% FCS (Gibco) and 100 U/mL penicillin and 100 ng/mL streptomycin.

### Lentiviral packaging and stable cell line establishment

The lentiviral packaging kit was purchased from Open Biosystems (Huntsville). Lentivirus carrying miR-124 or negative control (miR-NC) was packaged in 293T cells and collected from the supernatant as instructed by the manufacturer's manual. The lentiviruses were infected into MCF7 or MDA-MB-231 cells to establish stable cell lines, followed by puromycin selection.

### Isolation of RNA, reverse transcription PCR and quantitative real-time PCR

Total RNA was isolated from cultured cells or human tissues with TRIzol reagent according to the manufacturer's instruction (Invitrogen). To determine the quantity of the mRNA levels of AKT2, total RNAs were reverse transcribed by oligodeoxythymidine primer using the PrimeScript RT Reagent Kit. The housekeeping gene GAPDH was used as an internal control. The primers were as follows: AKT2 forward primer, 5′- ACCACAGTCATCGAGAGGACC -3′;AK T2 reverse primer, 5′-GGAGCCACACTTGTAGTCCA-3′; GAPDH forward primer, 5′-CCACCCATGGCAAATTCC ATGGCA-3′; GAPDH reverse primer, 5′-TCTAGACGGCA GGTCAGGTCCACC-3′. To measure the expression levels of miR-124, the stem-loop specific primer method was used as described previously [43, 44]. Quantitative reverse transcriptase (qRT) PCR primers were the following: miR-124 RT primer, 5′- CTCAACTGGTGTCGTGGAGTCGGCAATTCAGTTGAGGGCATTC-3′; miR-124 PCR primers, sense: 5′-ACACTCCAGCTGGGTAAGGCACGCGGTG-3′; antisense: 5′-TGGTGTCGTGGAGTCG-3′. U6 RT primer: 5′-AACGCTTCACGAATTTGCGT-3′; U6 PCR primers, sense: 5′-CTCGCTTCGGCAGCACA-3′; antisense: 5′-TGGTGTCGTGGAGTCG-3′. Quantitative RT-PCR was performed using SYBR Premix Dimer Eraser (Vazyme Biotech co., ltd) on a 7900HT system. GAPDH or U6 levels were used as an internal control, and fold changes were calculated by relative quantification (2^−ΔΔCt^).

### Luciferase reporter assay

The 3′-UTR-luciferase reporter constructs containing the 3′-UTR of AKT2 with wild-type and mutant binding sites of miR-124 were amplified using PCR method. The PCR products were cloned into the pMiR-luc reporter vector (Ambion) between SpeI and HindIII sites, immediately downstream of the luciferase gene. The mutant 3′-UTR constructs were made by introducing four mismatch mutations into the putative seed regions of AKT2. All the constructs containing 3′UTR inserts were sequenced and verified.

MDA-MB-231 cells (1.0×10^5^/well) were seeded in 24-well plates. After 24 h, cells were co-transfected with either wild-type (WT) or mutant-type (mut) luciferase reporter plasmids containing AKT2-3′-UTR, pGL4.74 vector control (Ambion) and equal amounts of pre-miR-124 or pre-miR-NC using Lipofectamine 2000 (Invitrogen) according the manufacturer's instruction. Luciferase activities were measured 24 h after transfection using the Dual Luciferase Reporter Assay System (Promega). Experiments were performed in triplicate with three independent replicates.

### Protein extraction and immunoblotting

Cells or tissues grounded in liquid nitrogen were lysed on ice for 30 min in radioimmunoprecipitation assay (RIPA) buffer (150 mM NaCl, 100 mM Tris, pH 8.0, 0.1% sodium dodecyl sulfate (SDS), 1% Triton X-100, 1% sodium deoxycholate, 5 mM EDTA, and 10 mM NaF) supplemented with 1 mM sodium vanadate, 2 mM aprotinin, 2 mM leupeptin, 1 mM phenylmethylsulfonyl fluoride, 1 mM dithiothreitol, and 2 mM pepstatin A. The lysates were centrifugated at 12,000 rpm at 4°C for 15 min, the supernatants were collected, and protein concentrations were determined using bicinchoninic acid assay. Protein extracts were separated by SDS–polyacrylamide gel electrophoresis and transferred to nitrocellulose membranes in transfer buffer (20 mM Tris, 150 mM glycine, 20% [volume/volume] methanol). Membranes were blocked with 5% nonfat dry milk for 2 h and incubated with primary antibodies (AKT2, Proteintech; GAPDH, Abcam). The protein bands were probed with secondary antibody, and visualized with the electrochemiluminescencedetection system (Thermo Scientific).

### Cell proliferation assay

Cells in the logarithmic phase of growth were seeded at 3,000/well and cultured in 96-well plates. Cell proliferation was assayed using the Cell-Counting Kit 8 (CCK8; Dojindo Laboratories) according to the manufacturer's instructions at indicated time points. Three independent experiments were performed in triplicate.

### Wound healing assay

Cells were cultured until reached 90% confluence in 6-well plates. Cell layers were scratched using a 10 μL tip to form wounded gaps, washed with PBS twice and cultured. The wounded gaps were photographed at different time points and analyzed by measuring the distance of migrating cells from five different areas for each wound.

### Invasion assays

Cell invasion was determined using 24-well invasion chambers with Matrigel (Becton Dickinson) according to the manufacturer's instructions. Cells (5×10^4^/well) were seeded in the upper well of the invasion chamber in DMEM without serum. The lower chamber well contained DMEM supplemented with 10% FBS to stimulate cell invasion. After incubation for 24 h, non-invading cells were removed from the top well with a cotton swab, while the bottom cells were fixed with 3% paraformaldehyde, stained with 0.1% crystal violet, and photographed in 3 independent 10× fields for each well. Membrane was air-dried and soaked for 15 min at room temperature with 33% acetic acid decolorization (200 μL/well). The destained solution was transferred to 96-well plates, and the absorbance value was read at an optical density of 570 nm. Three independent experiments were conducted in triplicate.

### Tumorigenesis in nude mice

For tumor growth assay, female nude mice [BALB/cA-nu (nu/nu), 6-wk-old] were purchased from Shanghai Laboratory Animal Center (Chinese Academy of Sciences) and maintained in special pathogen-free (SPF) condition for one week. Animal protocols were approved by the Animal Experimental Ethics Committee of Nanjing Medical University. MCF7 cells stably expressing miR-124 or miR-NC were injected subcutaneously into both flanks of nude mice (5×10^6^ cells in 100 μl serum-free DMEM medium). Tumor sizes were measured using vernier caliper every two days when the tumors were visible and tumor volume was calculated according to the formula: volume = 0.5×Length×Width^2^. Mice were sacrificed and tumors were dissected 17 days after implantation. Total proteins and RNAs were extracted for immunoblotting and qRT-PCR. Tumors were formalin-fixed, paraffin-embedded, and sectioned at 5μm for CD31 (Abcam) immunohistochemical staining under the standard procedure as previously described [45].

### Statistical analysis

Data in the present study were represented as means ± SD from at least three independent experiments except specific indicated. Student's unpaired *t* test was used for comparison between two groups. Data were analyzed with GraphPad Prism 5(La Jolla, CA, USA). For human tissue samples, AKT2 expression levels in adjacent normal and BC tissues were explored by Student's paired *t* test. AKT2 levels in ERα-positive and negative groups were analyzed using Student's unpaired *t* test. Values were considered significantly different when *P* < 0.05.

## SUPPLEMENTARY FIGURES


