# Supplementary material for: Estrogen regulates miRNA expression: implication of estrogen receptor and miR-124/AKT2 in tumor growth and angiogenesis
Source: Oncotarget. 2016 May 9;7(24):36940–55. doi: 10.18632/oncotarget.9230 (PMC5095050; doi:10.18632/oncotarget.9230)
Supplement: Supplementary file 1 [file oncotarget-07-36940-s001.pdf]

Estrogen regulates miRNA expression: implication of estrogen receptor and miR-124/AKT2 in tumor growth and angiogenesis

SUPPLEMENTARY FIGURES

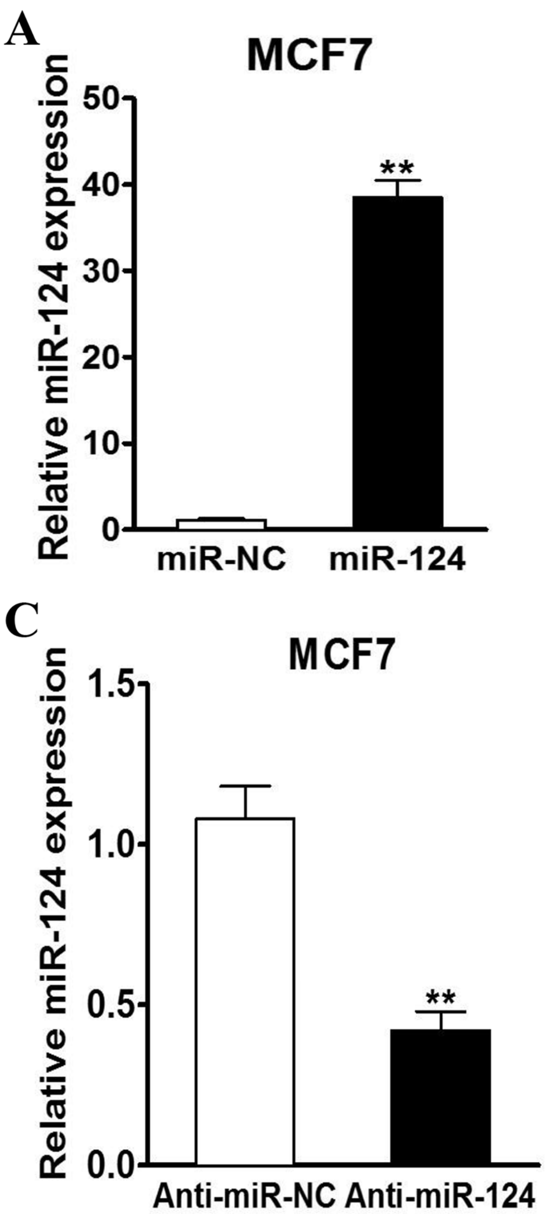

Supplementary Figure S1: The expression levels of miR-124 are significantly changed by miR-124 mimics or antagomirs in BC cells. A. and B. MCF7 and MDA-MB-231 cells were transfected with miR-124 mimics or miR-NC, **C. and D.** and miR-124 inhibitor (Anti-miR-124), or control anti-sense RNA inhibitor (Anti-miR-NC). The expression levels of miR-124 were analyzed by qRT-PCR and U6 levels were used as internal control, represented the ratio to control group. Data were presented as the means ± SD from three independent experiments with triple replicates per experiment. * indicates significant difference compared to control at $P<0.05$.

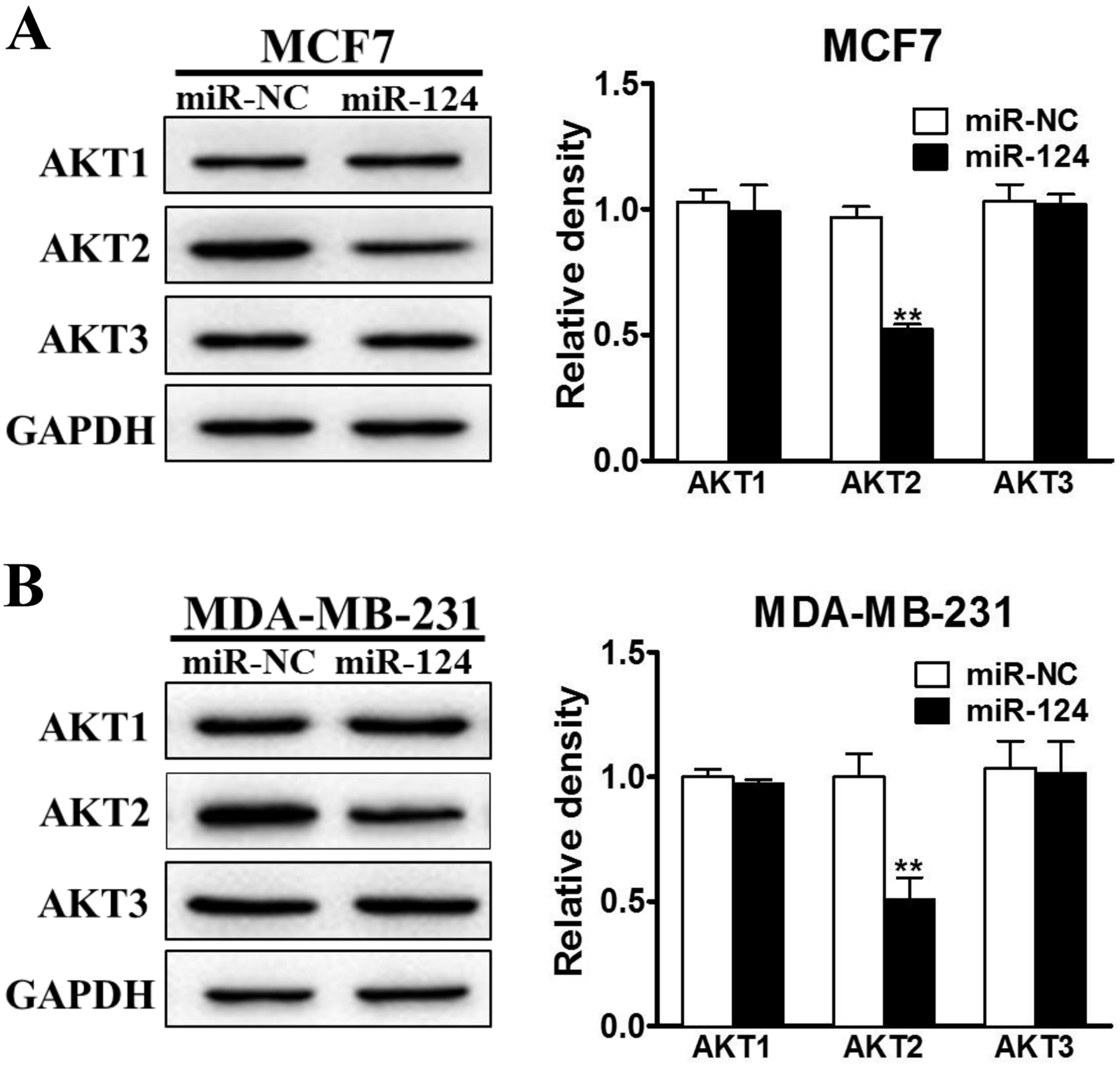

Supplementary Figure S2: miR-124 suppresses the expression levels of AKT2, but not AKT1 and AKT3. A. The expression levels of AKT1, AKT2, AKT3 and GAPDH were determined using immunoblotting in MCF7 and MDA-MB-231 cells overexpressing miR-124 and miR-NC. The density of protein levels of above was quantified by ImageJ software and GAPDH levels were used as internal control, and normalized to the values of miR-NC control. Data were presented as the means ± SD from three independent experiments with triple replicates per experiment. ** indicates significant difference compared to miR-NC group at $P < 0.01$.
